# Circulating Biomarkers in Localized Anal Squamous Cell Carcinoma Across Treatment Timepoints: A Systematic Review

**DOI:** 10.3390/cancers18101626

**Published:** 2026-05-18

**Authors:** Oluwatayo Adeoye, Abdulsabur Sanni, Khujasta Gul, Jakob Hamilton, Ahmed A. Abdelhakeem, Michael Rutenberg, Zhaohui Jin, Umair Majeed, Jeremy C. Jones, Conor D. O’Donnell

**Affiliations:** 1 Department of Hematology/Oncology, Mayo Clinic, Jacksonville, FL 32224, USA; adeoye.oluwatayo@mayo.edu (O.A.); gul.khujasta@mayo.edu (K.G.); abdelhakeem.ahmed@mayo.edu (A.A.A.); majeed.umair@mayo.edu (U.M.); jones.jeremy1@mayo.edu (J.C.J.); 2 Department of Hematology/Oncology, University of Minnesota, Minneapolis, MN 55455, USA; sanni027@umn.edu; 3 Department of Internal Medicine, Mayo Clinic, Jacksonville, FL 32224, USA; hamilton.jakob@mayo.edu; 4 Department of Radiation Oncology, Mayo Clinic, Jacksonville, FL 32224, USA; rutenberg.michael@mayo.edu; 5 Department of Oncology, Mayo Clinic, Rochester, MN 55905, USA; jin.zhaohui@mayo.edu

**Keywords:** anal squamous cell carcinoma, circulating biomarker, circulating tumor DNA, HPV DNA, tumor tissue-modified viral HPV DNA, minimal residual disease, chemoradiotherapy, liquid biopsy, surveillance, prognostic biomarker

## Abstract

**Background/Objectives:** Locoregional anal squamous cell carcinoma (ASCC) is usually cured with chemoradiotherapy; however, some patients relapse, require salvage abdominoperineal resection or develop metastatic disease. Approximately 90% of cases are driven by high-risk human papillomavirus, most commonly HPV16. Conventional surveillance using clinical examination and imaging may have limited sensitivity and specificity and is not individualized by recurrence risk. Circulating biomarkers (CBs), particularly circulating tumor DNA (ctDNA), have emerged as promising methods for real-time disease monitoring. We systematically reviewed available evidence evaluating CBs across treatment timepoints in localized ASCC. **Methods:** A PROSPERO-registered review (ID: 1133987) was conducted according to PRISMA guidelines. PubMed, EMBASE, Cochrane CENTRAL, Web of Science, and major conference proceedings (ASCO, ESMO, ASTRO) were searched on 30 November 2025. We included prospective or retrospective studies (≥10 patients) with stage I–III disease treated with curative-intent chemoradiotherapy that reported CBs, assay characteristics, and at least one clinical outcome. Studies with predominantly localized cohorts were included even if small proportions of metastatic patients were present, provided results relevant to curative-intent populations could be interpreted. Data were synthesized narratively by timepoint (baseline, mid-treatment, end of treatment, post-treatment/surveillance) and assay type given methodological heterogeneity. **Results:** Fifteen studies were included. CB assays comprised four categories: viral HPV ctDNA assays, tumor-informed ctDNA assays, non-specific total cell-free DNA (cfDNA) quantification, and circulating tumor cell (CTC)-based assays. Baseline detection rates varied by assay type. Viral HPV ctDNA assays demonstrated detection rates of 59–100%, while tumor-informed ctDNA assays showed rates of 79–89%. Across studies, higher CB detection rates and levels were generally associated with greater tumor burden, including more advanced T and N stage disease. Mid-treatment ctDNA clearance identified patients with excellent locoregional control and progression-free survival, whereas persistent ctDNA was associated with treatment failure. End-of-treatment and surveillance ctDNA positivity predicted recurrence within individual cohorts, with reported sensitivities of 80–90%, specificities of 95–99%, and molecular lead times preceding clinical or radiographic detection. In contrast, non-tumor-specific cfDNA dynamics showed more variable prognostic associations and were less consistently linked to tumor burden. **Conclusions:** Across heterogeneous assays, CB dynamics provide clinically meaningful prognostic information in localized ASCC, particularly when measured during treatment and early surveillance. Viral HPV and tumor-informed ctDNA may have the potential to guide follow-up intensity and inform future escalation or de-escalation strategies; however, prospective, standardized trials are needed to define actionable thresholds and test ctDNA-guided management.

## Simple Summary

Anal squamous cell carcinoma is rare, but more people are being diagnosed worldwide. Most patients are treated with chemoradiotherapy, yet some still experience regrowth of the cancer and need surgery that results in a permanent colostomy or develop metastatic disease. Currently, doctors rely on physical examinations and scans, which can sometimes be difficult to interpret after treatment. Scans have reported false-positive rates approaching 40%. Circulating tumor DNA (ctDNA) is made of tiny fragments of genetic material shed by cancer cells into the bloodstream and can be measured with a simple blood test. In this systematic review, we summarize all available studies evaluating circulating biomarkers (CBs) including ctDNA to monitor people with localized anal squamous cell cancer. Across 15 studies, these biomarkers, particularly ctDNA from viral and tumor-specific tests, demonstrate associations with treatment response and recurrence risk, supporting their potential to inform risk stratification and clinical management. Prospective, randomized studies with longer follow-up are needed to establish standardized thresholds, validate clinically actionable timepoints, and determine whether ctDNA-directed management can improve outcomes or reduce treatment-related burden in patients with ASCC.

## 1. Introduction

Anal squamous cell carcinoma (ASCC) is a rare disease [1], though incidence and mortality rates are rising globally [2] by approximately 2–3% per year [3,4]. Approximately 90% of cases are driven by high-risk human papillomavirus (HPV), most commonly HPV16 [5,6]. Both HPV-DNA and cell-free DNA shed from ASCC tumors cells may circulate in the blood as small fragments [7]. These fragments are referred to as circulating tumor DNA (ctDNA) and may be detectable through phlebotomy. Given that ctDNA has a very short half-life in circulation and can be detected at concentrations below 0.1 copies per milliliter with some assays, it offers great potential as a biomarker for detection and monitoring of ASCC during treatment and surveillance [8].

Approximately 79% of ASCC cases present as localized disease (stage I–III) [9]. Definitive chemoradiotherapy (CRT) is the recommended therapy by major guidelines including the National Comprehensive Cancer Network (NCCN) [9], American Society of Clinical Oncology (ASCO) [10], and American Society for Radiation Oncology (ASTRO) [11].

For patients who develop locoregional recurrence, salvage abdominoperineal resection (APR) is the standard recommended intervention. Up to 40% of individuals diagnosed with ASCC ultimately require a permanent colostomy within three years of their diagnosis [12,13,14]. Early identification of patients at risk for treatment failure remains a major unmet need.

Current NCCN surveillance recommendations are uniform and not adapted by recurrence risk. They rely on frequent clinical examinations—digital rectal exam and inguinal node palpation every 3–6 months for five years, anoscopy for three years, and annual cross-sectional imaging for stage II–III disease. Delayed tumor regression following CRT can make clinical response interpretation particularly difficult when determining when to intervene. Recommendations are generally to delay surgical assessment until at least 26 weeks after CRT in this context [9,15]. Current imaging modalities such as PET/CT/MRI do not accurately distinguish between residual disease and post-radiation inflammatory changes, with reported false-positive rates approaching 40%. Taken together, these surveillance methods contribute to unnecessary biopsies, patient anxiety, and added costs [12].

In recent studies, ctDNA has emerged as a promising noninvasive biomarker for real-time disease monitoring with the potential to overcome the limitations of current clinical assessment methods.

Many studies have focused on detecting circulating HPV DNA—most commonly HPV16 or HPV18—using droplet digital PCR (ddPCR) [16,17,18], quantitative PCR (qPCR) [19], or targeted next-generation sequencing (NGS) [12,20,21]. More recently, tumor-informed assays that track patient-specific somatic mutations, such as personalized multiplex PCR-NGS platforms (e.g., Signatera), have been applied in ASCC [12,20,21]. The term minimal residual disease (MRD) has been adopted to describe the detection of ctDNA in the absence of clinical/radiological evidence of disease during surveillance [8].

An initial systematic review by Temperley et al. [22], based on eight studies, concluded that ctDNA holds significant promise as a predictive and prognostic biomarker in ASCC but emphasized the need for standardized assays and larger validation cohorts. Since then, multiple additional retrospective and prospective studies have substantially expanded the evidence base worldwide, providing larger cohorts and more granular data on CB performance. Building on this progress, this systematic review includes all available studies through November 2025, encompassing 15 studies.

The primary aim is to evaluate the clinical relevance of CB (predominantly ctDNA) status at different timepoints—baseline, mid-treatment (MT), end of treatment (EOT), and post-treatment/surveillance—and to determine how CB dynamics correlate with treatment response, recurrence-free survival (RFS), disease-free survival (DFS), and overall survival (OS). We examine how ctDNA has been used to guide management in prospective clinical trials to date as well as outline current performance benchmarks that may inform endpoints for ctDNA-guided therapy escalation/de-escalation in future clinical trials.

## 2. Materials and Methods

### 2.1. Protocol and Registration

A review protocol was developed according to the Preferred Reporting Items for Systematic Reviews and Meta-Analyses (PRISMA) 2020 guidelines and registered with the International Prospective Register of Systematic Reviews (PROSPERO; Registration ID: 1133987) prior to study initiation. The protocol detailed the review of questions, background rationale, and methodology including inclusion criteria, search strategy, data extraction, and risk of bias assessment, summarized below.

### 2.2. Eligibility Criteria

Population: Patients with localized (non-metastatic) anal squamous cell carcinoma (Stage I–III) treated with curative-intent CRT were the focus. Studies needed to clearly distinguish this cohort (excluding purely metastatic or recurrent disease series). Studies with predominantly localized populations were included if small proportions of metastatic patients were present, particularly when outcomes were reported for or could be reasonably interpreted within curative-intent cohorts. Both prospective and retrospective studies were eligible.

Intervention (Biomarker): Assessment of circulating tumor DNA, via any platform detecting tumor-specific genetic material in blood, was considered: HPV-specific ctDNA assays (e.g., plasma HPV16/18 DNA by qPCR or digital PCR), Tumor Tissue-Modified Viral (TTMV) HPV DNA, Tumor-informed personalized ctDNA assays (e.g., NGS panels like Signatera), or broadly tumor-agnostic approaches (e.g., global cfDNA levels or methylation markers). Circulating tumor cell (CTC)-based assays were also included.

We included studies measuring CBs at one or more defined timepoints: pre-treatment/baseline, during CRT (e.g., midpoint), end of treatment (around completion of CRT, often 0–12 weeks post-therapy), and/or post-treatment surveillance (monitoring after initial complete response). Studies with serial CB measurements were evaluated for dynamic changes over time, whereas studies reporting CBs at a single timepoint were analyzed for cross-sectional prognostic or surveillance value without inference of kinetics.

Comparators: We considered comparisons of outcomes between patients with different CB statuses: CB-positive vs. CB-negative at baseline; persistence vs. clearance of CBs during or after treatment; and CB recurrence vs. no CB recurrence at follow-up.

Outcomes: Studies must report at least one clinical outcome in relation to CBs: treatment response (e.g., radiographic complete response vs. residual disease), recurrence (local and/or distant), disease-free survival (DFS) or relapse-free survival, overall survival (OS), or changes in management (e.g., early salvage interventions guided by CBs). Studies in which CBs directly informed treatment modification were analyzed descriptively to assess feasibility and clinical impact and were not used to evaluate CBs as independent prognostic biomarkers. Circulating viral DNA related to HPV-associated ASCC was interpreted separately to tumor-informed ctDNA, non-specific cfDNA and CTC.

Study Types: Given the rarity of anal cancer, all study designs were considered, including prospective cohort studies, retrospective analyses of prospectively collected samples, and relevant secondary analyses or pooled data. We also included abstracts from major conferences (ASCO, ESMO, ASTRO) if they provided sufficient data, to capture recent findings not yet in full publication. Case reports, review articles, editorials, and commentaries were excluded. Non-English language publications were excluded. We required a minimum sample size of ≥10 patients to focus on robust datasets.

### 2.3. Information Sources and Search Strategy

A comprehensive literature search was performed on 30 November 2025 across multiple databases: PubMed/MEDLINE, EMBASE, Cochrane CENTRAL, and Web of Science. The search string combined terms for anal cancer and ctDNA, including Medical Subject Headings (MeSH) and keywords: search strategy (“cell-free DNA” OR “circulating tumor DNA” OR “ctDNA” OR “liquid biopsy” OR “plasma DNA” OR “TTMV” OR “Tumor Tissue Modified HPV DNA”) AND (“anal cancer” OR “anal carcinoma” OR “anal neoplasm” OR “anal squamous cell carcinoma”) AND (“biomarker*” OR “diagnostic marker*” OR “predictive marker*” OR “prognostic marker*” OR “tumor marker*”) AND (“care” OR “intervention” OR “management” OR “therapy” OR “treatment”). No date restriction was imposed, and results were limited to English-language publications. We manually searched conference proceedings from ASCO, ESMO, and ASTRO to ensure inclusion of the most recent data. The reference lists of included studies and prior reviews (e.g., Temperley et al. 2024 [22]) were also cross-checked for any studies not captured by the database search.

### 2.4. Study Selection

**Figure 1:**
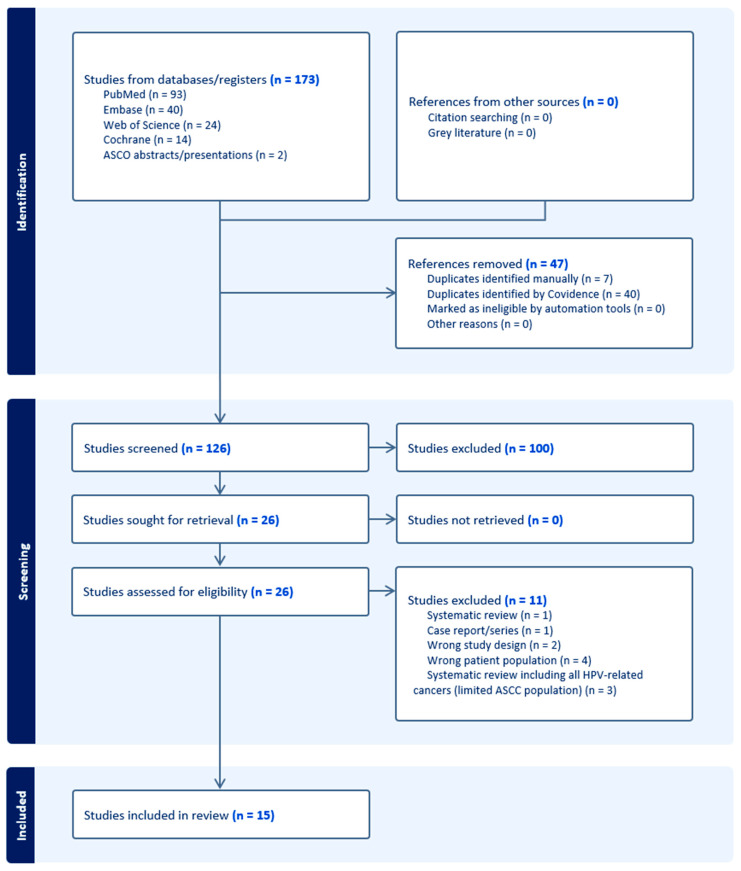
Selection of studies included in the systematic review (PRISMA).

All identified records were imported into Covidence systematic review software (Veritas Health Innovation, Melbourne, VIC, Australia; available at Covidence - Better systematic review management; accessed 30 November 2025), where duplicates were removed and study screening and data extraction were performed). Two independent reviewers (OA, AS) screened titles and abstracts for relevance. Studies clearly unrelated to anal cancer or not involving CBs were excluded at this stage. Full-text articles (or full conference abstracts) of remaining citations were then retrieved and assessed in detail against the eligibility criteria. Any disagreements on inclusion were resolved through discussion or by consulting a third reviewer (CO). A PRISMA flow diagram (Figure 1) was generated using Covidence systematic review software, detailing records identified, screened, assessed for eligibility, and included, with reasons for exclusion.

### 2.5. Data Extraction and Synthesis

A standardized data extraction form was used to collect key information from each included study—(1) study characteristics: first author, year, study design, sample size, and patient demographics; (2) disease specifics: tumor stage distribution (I–III) and HPV-status of tumors (if reported); (3) CB assay details: type of assay (e.g., HPV16-specific ddPCR, NGS-based panel, etc.), targeted analyte (viral DNA, somatic mutations, etc.), and blood sample timing (timepoints relative to treatment); (4) CB detection rates: proportion of CB-positive patients at baseline, fraction clearing during treatment, and CB-positive vs. -negative counts at end of treatment and specified follow-up intervals (e.g., 3, 6, 12 months); (5) outcome data: response rates (clinical complete response) in relation to CB status, recurrence rates or number of relapses among CB-positive vs. -negative groups, time to recurrence, survival outcomes (median DFS/OS if available, hazard ratios for CB associations), and any reported lead-time of CB detection ahead of clinical recurrence; (6) performance metrics: sensitivity, specificity, positive predictive value (PPV), and negative predictive value (NPV) of CBs (if reported) for outcomes like recurrence; (7) clinical utility: any instances of CBs influencing management (e.g., prompting earlier scans or interventions).

Given the expected heterogeneity in assays and endpoints, a narrative synthesis was planned. We grouped results primarily by timepoint of CB assessment (baseline, MT, EOT, surveillance) to facilitate cross-study comparison of findings at each interval. Where appropriate, ranges of values (e.g., detection rates, hazard ratios) are reported rather than attempting a meta-analysis. We refrained from pooling quantitative effect estimates due to differences in study populations and definitions (for example, what constituted “post-treatment” sampling varied between studies). Instead, we qualitatively describe consistent patterns and discrepant results. Where studies included mixed populations, interpretation focused on findings most applicable to curative-intent (stage I–III) disease.

### 2.6. Risk of Bias and Quality Assessment

The quality of each study was appraised using tools appropriate to its design. For observational cohort studies, the Newcastle–Ottawa Scale (NOS) was used, examining selection of participants, comparability of subgroups, and outcome assessment. Overall, 11 studies were assessed as having low risk of bias and 4 as having moderate risk of bias based on NOS criteria, with no studies classified as high-risk (Supplementary Table S1). Key potential biases included patient selection bias (e.g., inclusion of only good responders), measurement bias in CB assays (e.g., differing thresholds), and incomplete follow-up. Conference abstracts (*n* = 3 studies) were inherently limited by less detail; we noted these as preliminary evidence. No studies were randomized (no intervention beyond standard care), so traditional trial risk-of-bias tools did not apply. We did not formally exclude any study based on quality, but study limitations are considered in interpreting results. Overall, most included studies were single-center cohorts, often exploratory in nature, which we account for in our discussion of evidence of strength.

## 3. Results

### 3.1. Study Characteristics

We evaluated 15 studies. Most of the cohorts consisted of individuals diagnosed with localized ASCC, primarily stage I–III, who received definitive CRT. The median patient age was in the mid-60s. High-risk HPV infection was nearly universal across studies, with most tumors demonstrating HPV or p16 positivity. Across studies, median follow-up ranged from approximately 1 to 3 years.

### 3.2. Assay Platforms

Assay platforms varied across studies and reflected distinct biological targets (Table 1a–d). Viral HPV ctDNA assays were most common (*n* = 8 studies), followed by tumor-informed ctDNA assays targeting patient-specific somatic mutations (*n* = 3 studies), non-specific total cell-free DNA (cfDNA) quantification (*n* = 3 studies), and circulating tumor cell (CTC)-based assays (*n* = 1 study). This heterogeneity reflects the different biological approaches used to detect tumor-derived material in the circulation (Supplementary Table S2).

**Table 1:** (a): Study Characteristics—Viral HPV ctDNA (n = 444) *. (b): Study Characteristics—Tumor-Informed ctDNA/Signatera (n = 125) *. (c): Study Characteristics—Total cfDNA (Not Viral or Tumor-Informed; n = 232). (d): Study Characteristics—Circulating Tumor Cells (n = 15).

**(a)**								
**Study (Year)**	**N**	**Median Age (Range)**	**Sex (F/M)**	**Stage**	**HPV+/−**	**HIV+/−**	**Assay**	**Specimen**
Cabel (2018) [23]	33	64 (47–82)	29/4	Stage II (33%), IIIA (27%), IIIB (40%)	HPV 16+ (31); HPV 18+ (2)	3+/30−	ddPCR (HPV16/18 E7)	Plasma
Lee (2020) [24]	24 (21 analyzed)	NR	12/9	Stage I–II (43%) III (57%)	20+/1 HPV− (E7 mRNA analyses)	4+/17−	HPV-targeted NGS (panHPV-detect)	Plasma
Lefevre (2021) [16]	88	63 (26–84)	65/23	T1 (22%), T2 (60%), T3 (10%), T4 (8%); N0 (76%), N1 (24%); M0 (99%), M+ (1%)	p16+ (72)/p16− (13); Unknown (3)	NR	ddPCR (HPV16/18/31/33/51/58)	Plasma
Mazurek (2023) [19]	62 (35 tested)	63 (19–83)	49/13	T1 (9%), T2 (41%), T3 (40%), T4 (10%); N0 (59%), N1a (28%), N1c (13%)	27+/8 ctHPV16− (no direct tissue assessment); 27 without pretreatment testing	NR	qPCR ctHPV16 (TaqMan)	Plasma
Morris (2025) [25]	65	62.8 (43–84)	50/15	Stage I–II (55.4%), III (44.6%)	51+/1− (13 unknown)	NR	ddPCR (13 HR-HPV types)	Plasma
Kim (2025) [26]	55	63.9 (IQR 57.1–72.3)	42/13	T1 (2%), T2 (15%), T3 (44%), T4 (36%), TX (4%); N0 (10%), N1 (73%), N1c + M1a (4%), NX (5%)	41+/9− HPV ctDNA; 5 unknown	NR (excluded if CD4 count <400/mm^3^)	Digital PCR HPV ctDNA	Plasma
Agarwal (2025) [18] †	13 (11 analyzed)	63 (NR)	NR	Stage IIA (9.1%), IIB (64%), IIIA (9.1%), IIIB (18.1%)	11 HPV+	NR	TTMV-HPV DNA ddPCR	Plasma
Kabarriti (2025) [17]	117 (80 TTMV-HPV DNA+)	63 (36–91)	85/32	Stage I (10%), II (26%), III (58%), IV (6%)	HPV 16+ (74), HPV 16/18+ (1), HPV 18+ (3), HPV 33+ (2)	14+/93− (10 unknown)	TTMV-HPV DNA ddPCR	Plasma
**(b)**								
Azzi (2023) [12]	251 (37 analyzed)	63.5 (27.9–89.4)	180/71 (19/18 analyzed)	Stage I (8%), II (27%), III (46%), IV (19%)	20+/1 HPV−; Unknown (16)	12+/19−: Unknown (6)	Tumor-informed NGS ctDNA (Signatera)	Plasma
Alvarez (2023) [20] †	41	NR	NR	Stage I–III (66% stage III)	NR	NR	Tumor-informed NGS ctDNA (Signatera)	Plasma
Bercz (2025) [21] †	88	NR	NR	Localized ASCC	NR	NR	Tumor-informed NGS ctDNA (Signatera)	Plasma
**(c)**								
Lefevre (2020) [27]	80	63 (26–84)	61/19	T1 (19%), T2 (59%), T3 (10%), T4 (12%); N0 (74%), N+ (26%); M0 (99%), M1 (1%)	55+/7 HPV−: Unknown (18)	NR	DFA for total cfDNA	Serum
Małusecka (2022) [28]	26	NR	21/5	T1 (15%), T2 (54%), T3 (23%), T4 (8%); N0 (42%); N+ (58%)	NR	NR	qPCR (TERT amplification) for total cfDNA	Plasma
Jakobsen (2025) [29]	126	67 (18–86)	92/34	T1 (13%), T2 (55%), T3 (15%), T4 (17%); N0 (63%), N+ (37%); M0 (98%), M+ (2%)	113+/13 p16−	NR	DFA for total cfDNA	Serum
**(d)**								
Ruano (2023) [13]	15	61 (43–73)	10/5	Stage III (73%); cN1 (67%)	14+/1 HPV−	0+/15−	CTC-based assay with HPV DNA detection	Whole blood

Abbreviations: AJCC, American Joint Committee on Cancer; ASCC, anal squamous cell carcinoma; cfDNA, cell-free DNA; CTC, circulating tumor cell; ctDNA, circulating tumor DNA; ddPCR, droplet digital polymerase chain reaction; DFA, direct fluorescent assay; HIV, human immunodeficiency virus; HPV, human papillomavirus; HR-HPV, high-risk human papillomavirus; IHC, immunohistochemistry; IQR, interquartile range; NGS, next-generation sequencing; NR, not reported; PCR, polymerase chain reaction; qPCR, quantitative polymerase chain reaction; TTMV-HPV DNA, tumor tissue-modified viral HPV DNA. † Conference abstract. * Some cohorts overlap. Agarwal is a subset of Kabarriti, and Alvarez likely overlaps with Bercz. Studies are listed separately for distinct analyses; patient counts should not be considered independent.

Tumor-informed multiplex PCR–next-generation sequencing (mPCR-NGS; Signatera^®^) was used in studies by Azzi [12], Alvarez [20] and Bercz [21]. The Alvarez patient cohort likely represents a subset of the Bercz study [20,21]. Quantitative results expressed as mean tumor molecules per mL (MTM/mL) were reported only in Alvarez [20]. Several studies applied droplet digital PCR (ddPCR) assays targeting HPV E6/E7 genes. Cabel [23] measured circulating HPV16 and HPV18 ctDNA in patients with advanced or locally advanced disease. Lefèvre [16] and Morris [25] used ddPCR panels to detect multiple high-risk HPV subtypes including HPV16, 18, 31, 33, 35, 45, 52, and 58. Kabarriti [17] and Agarwal [18] used the NavDx^®^ assay, a multianalyte ddPCR platform quantifying tumor tissue-modified viral (TTMV) HPV DNA fragments derived from five high-risk subtypes (HPV16, 18, 31, 33, and 35). The Agarwal patient cohort represents a subset of the population included in the Kabarriti study [17,18]. Kim et al. [26] utilized ddPCR to measure HPV ctDNA, with a detection cutoff set at 20 DNA copies/mL. Mazurek [19] used quantitative PCR (qPCR) to quantify circulating HPV16 DNA load in plasma, correlating viral copy numbers with tumor burden and lymph node involvement. The panHPV-detect assay described by Lee [24] utilized targeted NGS amplification of eight HPV types (HPV16, 18, 31, 33, 35, 45, 52, and 58) and defined ctDNA positivity by a threshold of ≥5.5 viral amplicons. Three studies analyzed total cell-free DNA (cfDNA) without tumor or viral specificity. Jakobsen [29] and Lefèvre [27] applied a direct fluorescent assay (DFA) quantifying cfDNA concentration in ng/µL, while Małusecka [28] performed qPCR targeting the telomerase reverse transcriptase (TERT) gene. Ruano [13] uniquely investigated circulating tumor cells (CTCs) using chromogenic in situ hybridization to detect HPV DNA within isolated CTCs.

Given these fundamental biological differences, results from viral ctDNA assays, tumor-informed ctDNA assays, and non-specific cfDNA or CTC measurements were not pooled or compared quantitatively across platforms and were interpreted within their respective biological contexts. Throughout the “Results”, viral ctDNA refers to circulating HPV DNA detected in plasma using the associated platforms described above.

### 3.3. Baseline CBs and Associations with Tumor Burden (Table 2a–d)

Baseline tumor-specific ctDNA positivity ranged from 59% (52/88) in Lefevre et al. [16] to 100% in selected HPV-restricted cohorts (e.g., 20/20 in Lee et al.) [24]. Tumor-informed ctDNA assays similarly demonstrated high baseline detection, with positivity rates of 79% in Bercz et al. [21] and 89% in Alvarez et al. [20]. In contrast, studies quantifying total cfDNA without tumor or viral specificity (Jakobsen et al. [29]) reported universal baseline detectability, reflecting the non-specific presence of circulating DNA rather than tumor-derived ctDNA.

**Table 2:** (a): Baseline Viral HPV ctDNA Positivity and Correlation with Tumor Burden. (b): Baseline Tumor-Informed ctDNA and Correlation with Tumor Burden. (c): Baseline cfDNA Positivity and Correlation with Tumor Burden. (d): Baseline CTCs and Correlation with Tumor Burden.

**(a)**			
**Study** **(Year)**	**Detection Rate % (** ***n*** **)**	**Notes**	**Correlation with Disease Burden**
Cabel (2018) [23]	88% (29/33)	Sensitivity increased from 64% in stage II (95% CI 35–85) to 100% in stage III patients (95% CI 85–100; *p* = 0.008). Higher baseline concentrations N+ vs. N0 (85.5 vs. 32 copies/mL, *p* = 0.03)	Yes
Lee (2020) [24]	100% (20/20) HPV+ ASCC; 0/21 HPV− controls	cHPV-DNA detected in all 20 HPV+ patients and 0/21 HPV− controls (95% CI 83–100%). No relationship found between ctDNA levels and tumor volume (r^2^ = 0.3, *p* = 0.13)	No
Lefèvre (2021) [16]	59% (52/88)	Median fractions increased from T1 (0.35%) to T4 (13.46%); six-fold higher in N+ vs. N0 (6.09% vs. 0.39%, *p* = 0.02). Higher baseline pHPV (≥1.34%) trended toward inferior DFS (HR 4.07, 95% CI 0.84–19.64, *p* = 0.08) and OS (HR 2.42, 95% CI 0.44–13.44, *p* = 0.31)	Yes
Mazurek (2023) [19]	77% (27/35)	Median viral load (844 copies/mL; range 6–31,500; log_10_ range 0.8–4.5). Higher baseline viral loads correlated significantly with N+ (*p* = 0.031)	Yes
Agarwal (2025) [18]	85% (11/13)	No association with stage, clearance during CRT, or recurrence.	No
Kabarriti (2025) [17]	85.4% (41/48)	Sensitivity increased from N0 (76.5%, 13/17; 95% CI 74.9–95.3) to N+ (90.3%, 28/31; 95% CI 75.4–95.4).	Yes
Morris (2025) [25]	68% (44/65)	Median ctDNA level (139.4 copies/mL; range 18.9–26,770) correlated significantly with higher T stage (T3–T4; OR 5.1, *p* = 0.01), N+ (OR 4.3, *p* = 0.01), and stage III disease (OR 5.6, *p* = 0.007)	Yes
Kim (2025) [26]	74% (40/54)	NR	NR
**(b)**			
Alvarez (2023) [20]	89% (32/36)	Higher baseline ctDNA levels in stage III vs. stage I–II (median 29 vs. 2.9 MTM/mL, *p* = 0.04)	Yes
Bercz (2025) [21]	79% (61/77)	NR	NR
**(c)**			
Lefèvre (2020) [27]	Median 0.92 ng/µL (95% CI 0.88–0.99)	Positive correlation with tumor burden, including gross tumor volume (R^2^ = 0.13, *p* < 0.01) and increasing T stage (T1 0.80; T2 0.94; T3 1.11; T4 1.30 ng/µL)	Yes
Małusecka (2022) [28]	4.88–49.0 ng/mL (median 9.95 ng/mL)	No correlation between cfDNA levels and T stage or nodal status. Correlation observed with primary tumor volume on PET/CT (r = 0.9, *p* = 0.00006).	Yes
Jakobsen (2025) [29]	100% (126/126)	cfDNA concentrations were highest in T3 tumors (*p* = 0.05); T4 tumors showed elevated but non-significant differences compared with T1-T2 (*p* = 0.80). No association with N (*p* = 0.44)/M status (*p* = 0.90). When grouped by composite risk, cfDNA levels were significantly higher in high-risk disease (T3–T4/N+ or M+) compared with low-risk disease (T1–T2N0M0; *p* = 0.005)	Yes
**(d)**			
Ruano (2023) [13]	100% (15/15) CTCs detected (median 0.4 CTCs/mL, range 0.4–3.33) HPV DNA detected in 14/15 (93.3%) CTC samples.		NR

Abbreviations: ASCC, anal squamous cell carcinoma; cfDNA, cell-free DNA; CI, confidence interval; CRT, chemoradiotherapy; CTC, circulating tumor cell; ctDNA, circulating tumor DNA; DFS, disease-free survival; HPV, human papillomavirus; HR, hazard ratio; N+, node-positive disease; N0, node-negative disease; NR, not reported; OR, odds ratio; OS, overall survival; PET/CT, positron emission tomography/computed tomography.

In Kabarriti’s NavDx (TTMV-HPV DNA) cohort, pretreatment detection was 85.4% (41/48); baseline detection increased from 76.5% in node-negative cases (13/17, 95% CI 74.9–95.3) to 90.3% in node-positive disease (28/31, 95% CI 75.4–95.4) [17].

Agarwal’s study identified ctDNA in 11 out of 13 (85%) HPV-positive patients, but did not establish any correlation with disease stage, clearance rate, or recurrence probability [18].

Cabel et al. reported baseline detection rates of 88% (29/33). They identified HPV ctDNA in 64% of patients with stage II (95% CI 35–85) compared to 100% of stage III patients (95% CI 85–100; *p* = 0.008), with median baseline concentrations being greater in node-positive cases than in node-negative cases (85.5 vs. 32 copies/mL, *p* = 0.03) [23].

Lefèvre et al. reported HPV DNA in 52/88 (59%) patients, with median fractions rising from 0.35% in T1 disease to 13.46% in T4 disease and being six-fold higher in node-positive vs. node-negative tumors (6.09% vs. 0.39%, *p* = 0.02). Higher baseline pHPV (≥1.34%) trended toward inferior DFS (HR 4.07, 95% CI 0.84–19.64, *p* = 0.08) and OS (HR 2.42, 95% CI 0.44–13.44, *p* = 0.31) [16].

Mazurek et al. reported baseline circulating HPV16 DNA positivity in 27/35 (77%) patients. Eight patients (23%) were negative. The median viral load was 844 copies/mL (range 6–31,500; log_10_ range 0.8–4.5), and higher baseline viral loads correlated significantly with nodal positivity (*p* = 0.031) [19].

Morris et al. reported that HPV ctDNA was detected in 44 of 65 patients (68%). The median ctDNA level was 139.4 copies/mL (range 18.9–26,770). Baseline viral ctDNA positivity correlated significantly with higher tumor stage (T3–T4; OR 5.1, *p* = 0.01), nodal involvement (OR 4.3, *p* = 0.01), and stage III disease (OR 5.6, *p* = 0.007) [25].

Lee’s panHPV-detect NGS assay demonstrated 100% baseline sensitivity and specificity for distinguishing HPV-positive ASCC from HPV-negative controls (95% CI 83–100%). No relationship was found between circulating HPV DNA signal and tumor volume (r^2^ = 0.3, *p* = 0.13) [24].

In the phase-2 INTERACT-ION trial, ddPCR detected HPV ctDNA in 75% of patients (41/55 or 74% in the modified intention-to-treat population) at baseline. In total, 80% of patients had T3–T4 disease, and 76% of patients had nodal involvement given that this was a prospective trial in the locally advanced disease setting. No correlation analyses were provided regarding the relationship between baseline ctDNA status and T stage or N stage [26].

Alvarez et al. reported a baseline positivity of 89% (32/36), with higher baseline ctDNA levels in stage III vs. stage I–II disease (median 29 vs. 2.9 MTM/mL, *p* = 0.04) with tumor-informed assays [20].

Azzi et al. reported higher ctDNA positivity and levels in metastatic vs. localized disease (71.4% vs. 40.6%, *p* = 0.0002; *p* = 0.004) with tumor-informed assays, with no significant differences among stage I–III disease [12].

Jakobsen et al. reported universal baseline cfDNA detectability (126/126). p16 overexpression was present in 90% of evaluable tumors, and baseline cfDNA levels did not differ by p16 status. cfDNA concentrations were highest in T3 tumors (*p* = 0.05), while elevations in T4 tumors were not significantly different from T1–T2 (*p* = 0.80). There was no association between baseline cfDNA and nodal (*p* = 0.44) or metastatic status (*p* = 0.90). However, when applying a composite risk definition, baseline cfDNA levels were significantly higher in high-risk disease (T3–T4, N^+^, or M^+^) compared with low-risk disease (T1-T2N0M0; *p* = 0.005) [29].

Lefèvre et al. reported a median baseline cfDNA level of 0.92 ng/µL (95% CI 0.88–0.99) in 73 samples and observed a positive correlation with tumor burden, including gross tumor volume (R^2^ = 0.13, *p* < 0.01) and increasing T stage (T1 0.80; T2 0.94; T3 1.11; T4 1.30 ng/µL) [27].

In contrast, Małusecka et al. reported pre-treatment cfDNA concentrations ranging from 4.88 to 49.0 ng/mL (median 9.95 ng/mL) and found no correlation between cfDNA levels and T stage or nodal status. Baseline cfDNA levels were numerically higher in patients with progressive disease compared with those achieving partial or complete response (19.95 vs. 9.95 ng/mL), although this difference was not statistically significant [28].

Ruano et al. detected baseline CTCs in all patients (15/15), with a median of 0.4 CTC/mL (range 0.4–3.33). HPV DNA was detected in CTCs in 14/15 patients (93.3%) at baseline. All primary tumors were p16-positive by immunohistochemistry. No association between baseline CTC levels and tumor burden was reported [13].

### 3.4. Mid-Treatment CBs and Outcomes (Table 3a–c)

Across studies, mid-treatment clearance of viral or tumor-informed ctDNA was associated with improved outcomes, whereas cfDNA kinetics showed more variable associations.

**Table 3:** (a): Viral HPV ctDNA Dynamics Across Treatment Timepoints and Associated Outcomes. (b): Tumor-Informed ctDNA Dynamics Across Treatment Timepoints and Associated Outcomes. (c): cfDNA Dynamics Across Treatment Timepoints and Associated Outcomes.

**(a)**				
**Study (Year)**	**MT**	**EOT**	**Post-Treatment/Surveillance**	**Median Follow-Up in Months (Range)**
Cabel (2018) [23]	NR	3/18 (17%) ctDNA+ → 100% with early metastatic relapse at 2.9, 3.8, and 4.3 months; ctDNA− in 15/18 (83%) → only 1/15 (7%) recurred (local, 8.5 months); shorter DFS (*p* < 0.0001) if residual positivity at EOT.	9 ctDNA− samples at 90–150 days post-CRT → 0 recurrences.	30 (8–60)
Lee (2020) [24]	7/8 patients ctDNA− at 6 w	15/17 (88%) ctDNA− at 12 w. 2 patients (12%) remained ctDNA+ at 12 w → both relapsed (1 local, 1 distant). Sensitivity 100%, Specificity 100% for relapse prediction at 12w.	1 patient with persistent ctDNA+ despite cCR → distant relapse at 9 months. 4 patients with MRI abnormalities but ctDNA− remained disease-free on PET/MRI at 6–12 months.	16.8 (4–24)
Lefèvre (2021) [16]	12/45 “fast responders” (pHPV− by MT) → no local/distant treatment failures	20/45 “slow responders” pHPV− by EOT → 20% local, 0% distant relapse. 13/45 “persistent +” → 31% distant, 0% local relapse Patients with distant failures had median EOT pHPV of 0.12%, increasing to 13.96% at recurrence.	22 pHPV− in initial sample → 4/22 (18%) developed local recurrence.	NR
Mazurek (2023) [19]	8/10 (80%) ctHPV16 clearance during CRT → all with remission	ctHPV16− in most patients after CRT.	ctHPV16 detection during surveillance corresponded with recurrence	NR
Agarwal (2025) [18]	5/11 (45%) cleared at week 4; 4/5 remained disease-free vs. 3/6 (50%) with persistent ctDNA relapsed (1 local persistence at 1 month, 1 combined local and distant recurrence at 6 months, and 1 distant recurrence at 12 months)	1/6 ctDNA− developed recurrence. 4/11 patients with residual/recurrent disease → 3/4 ctDNA+ by EOT (suggested sensitivity of 75%).	1 mo post CRT (10): ctDNA− (8); persistent/recurrent ctDNA+ (2) → distant recurrence (1) and local and distant recurrence (1)	13
Kabarriti (2025) [17]	NR	19/25 (76%) cleared TTMV-HPV DNA during or within 3 months of CRT → better RFS (*p* = 0.0099).	88.9% (104/117) underwent testing → 21.2% with at least 1 positive result → 26% (27/117) clinical recurrence (14 local, 13 distant). Assay performance (per-test): sensitivity 85.4%, specificity 99.3%, PPV 97.6%, NPV 95.0%. ctDNA+ preceded clinical/radiologic recurrence in 58.3%. Median lead time: 59 days (range 10–536). Testing resolved 94.3% of indeterminate imaging/clinical findings.	19 (24 in post-treatment subset)
Morris (2025) [25]	NR	EOT ctDNA not prognostic: HR 1.6 (95% CI 0.35–7.4; *p* = 0.48).	Relapse occurred in 80% of ctDNA+ vs. 2% of ctDNA− patients (OR 168; *p* < 0.0001). Median RFS 4.9 mo for ctDNA+ vs. NR in ctDNA− (HR 39.2, 95% CI 4.6–330; *p* < 0.0001); 6 mo median RFS 5.6 mo vs. NR (HR 32.0, 95% CI 1.8–560; *p* < 0.0001). 89% sensitivity, 95% specificity, 80% PPV, and 98% NPV. 2 patients with recurrence despite negative ctDNA at 3 mo → converted to positive at later timepoints.	16.3 (7.1–26.8)
Kim (2025) [26]	90% (36/40) achieved BCR at 8 weeks BCR + ≥30% radiographic response (RECIST) + pathologic CR/near-CR (<10% viable tumor) → INRT (75%; 38/51)	Clinical CR at 40 weeks → INRT: 86.8% (90% CI 74.3–94.7; n = 33); standard CRT: 69.2% (90% CI 42.7–88.7; n = 9); overall: 77.8% (42/54; 90% CI 66.5–86.7). PFS → INRT: 92.1% (12 mo), 89.4% (24 mo); standard CRT: 84.6% (12 mo), 76.9% (24 mo).	NR	23 (16.5–29.1)
**(b)**				
Azzi (2023) [12]	NR	22/23 ctDNA− → no recurrence; 1 biopsy-proven residual disease (NPV 95.7%). 3/4 ctDNA+ → progression on imaging; 1 with partial radiologic response.	Post-treatment ctDNA+ strongly associated with inferior DFS: median DFS 11.4 mo vs. NR; HR 28.0 (95% CI 2.8–285.0; *p* = 0.005). During surveillance, ctDNA+ in 43.2% (16/37) of patients. One case with molecular recurrence preceding radiographic recurrence by 200 days.	21.0 (4.1–67.3)
Alvarez (2023) [20]	58% (14/24) cleared	ctDNA decreased from 23 to 0.01 MTM/mL (*p* = 0.01); 95% of patients ctDNA- by treatment completion.	Median time to ctDNA clearance 31 days vs. 131 days to cCR (*p* < 0.0001).	5.5 (0–22.3)
Bercz (2025) [21]	53% (33/62) clearance → 0% LRF and 100% 1-yr PFS; persistent ctDNA+ → 26% LRF	6/73 (8%) had persistent ctDNA post-CRT → 61% LRF and one-year PFS of 44%.	Assay with 100% sensitivity for recurrence in surveillance (predated clinical/radiographic recurrence in all cases).	18 (IQR 11–26)
**(c)**				
Lefèvre (2020) [27]	Decreased baseline → MT (0.92 → 0.78 ng/µL, *p* < 0.01); not prognostic	Increased MT → EOT (0.78 → 0.89 ng/µL, *p* < 0.01); higher with radiation dermatitis (*p* = 0.04).	Declined at 1 year (0.71 ng/µL); cfDNA kinetics not associated with recurrence.	22
Małusecka (2022) [28]	cfDNA increased during CRT; similar kinetics across stages; higher peak levels in T3–T4 vs. T1–T2 tumors (*p* < 0.01); no association with treatment response	Peak cfDNA levels observed at EOT.	cfDNA declined during follow-up and fell below baseline after >2 years; not associated with survival outcomes.	30
Jakobsen (2025) [29]	A ≤20% decline in cfDNA during CRT → worse DFS (HR 3.13, *p* = 0.01; AUC = 0.70)	NR	NR	22.2

Abbreviations: cfDNA, cell-free DNA; cHPV, circulating human papillomavirus DNA; CI, confidence interval; cCR, clinical complete response; CRT, chemoradiotherapy; ctDNA, circulating tumor DNA; CTC, circulating tumor cell; ddPCR, droplet digital polymerase chain reaction; DFA, direct fluorescent assay; DFS, disease-free survival; EOT, end of treatment; HPV, human papillomavirus; HR, hazard ratio; IQR, interquartile range; LRF, locoregional failure; MT, mid-treatment; NGS, next-generation sequencing; NR, not reported; OR, odds ratio; PCR, polymerase chain reaction; PFS, progression-free survival; PPV, positive predictive value; NPV, negative predictive value; qPCR, quantitative polymerase chain reaction; RECIST, Response Evaluation Criteria in Solid Tumors; TERT, telomerase reverse transcriptase; TTMV-HPV DNA, tumor tissue-modified viral human papillomavirus DNA. → indicates the subsequent clinical outcome or next observed event.

Bercz et al. observed ctDNA clearance in 33/62 (53%) patients by mid-treatment; this group had 0% locoregional failures and 100% one-year PFS, whereas persistent ctDNA was associated with a 26% locoregional failure rate and 81% one-year PFS [21].

Alvarez et al. reported ctDNA clearance in 14/24 (58%) baseline-positive patients during CRT. ctDNA clearance occurred significantly earlier than clinical complete response (cCR), with a median time to ctDNA clearance of 31 days vs. 131 days to cCR (*p* < 0.0001) [20].

Agarwal et al. observed that at mid-treatment (week 4), 5 of 11 patients (45%) achieved ctDNA clearance. Of these, four remained disease-free and one developed distant metastasis. The remaining six patients (55%) had persistent ctDNA at week 4; within this group, three experienced treatment failure (one with local persistence at 1 month, one with combined local and distant recurrence at 6 months, and one with distant recurrence at 12 months) [18].

Lefèvre et al. reported outcomes based on distinct ctDNA clearance patterns. Fast molecular responders (n = 12) cleared plasma HPV DNA (pHPV) by MT and experienced no local or distant failures. Slow responders (n = 20) achieved clearance only by EOT, with a 20% rate of local failure but no distant relapse [16].

Kim et al. reassessed HPV ctDNA at 8 weeks following induction chemo-immunotherapy. Following induction, 36 of 40 (90%) patients with baseline ctDNA positivity achieved biological complete response (BCR), defined as a reduction in HPV ctDNA to <20 copies/mL. Patients were eligible for de-escalated radiation [involved-node chemoradiation (INRT)] if they met all three criteria after induction: (1) BCR; (2) major radiographic response [≥30% tumor reduction per Response Evaluation Criteria in Solid Tumors (RECIST)]; and (3) pathological complete or near-complete response (<10% viable tumor cells on biopsy). Because ctDNA dynamics contributed to eligibility for treatment de-escalation, outcomes are presented descriptively and not interpreted as independent prognostic validation. These criteria were met by 38 of 51 (75%) patients who proceeded to INRT, while 13 of 51 (26%) patients proceeded to standard CRT. Although outcomes were not stratified by ctDNA status alone, clinical complete response rates at 40 weeks (26 weeks after CRT) were 86.8% (90% CI 74.3–94.7) among patients treated with INRT (n = 33) and 69.2% (90% CI 42.7–88.7) among those receiving standard CRT (n = 9). With an overall clinical complete response rate of 77.8% (42/54; 90% CI 66.5–86.7), the study met its primary endpoint of improved clinical complete response compared to historical control using a strategy that involved early dynamics for ctDNA to guide de-escalation of radiation. With a median follow-up of 23 months, PFS at 12 and 24 months was 92.1% and 89.4% for the INRT group, compared to 84.6% and 76.9% for the standard CRT group, respectively. Recurrence occurred in 4 of 38 patients (11%) treated with INRT and 4 of 13 (31%) receiving standard CRT. In post hoc analysis, recurrences in the INRT group involved the liver, lung, rectum, and subcutaneous tissue, while those in the standard CRT group occurred in lymph nodes (n = 2), the subscapularis muscle (n = 1), and combined anal plus nodal sites (n = 1) [26].

Mazurek et al. reported that 8 of 10 patients (80%) achieved ctHPV16 clearance during chemoradiotherapy and subsequently achieved complete remission, whereas persistent ctHPV16 during treatment was associated with disease progression and death in one patient [19].

Jakobsen et al. reported a decline in median cfDNA concentration from 0.78 ng/µL (95% CI 0.72–0.85) at baseline to 0.62 ng/µL (95% CI 0.56–0.72) at mid-treatment. A ≤ 20% decline in cfDNA during CRT was associated with significantly worse disease-free survival (HR 3.13, *p* = 0.01; AUC = 0.70) [29].

Małusecka et al. reported cfDNA levels increased during CRT compared with baseline and followed a similar kinetic pattern across tumor stages. Peak cfDNA levels during treatment were significantly higher in patients with T3–T4 tumors compared with T1–T2 tumors (*p* < 0.01), while lymph node status did not influence cfDNA kinetics (*p* = 0.79) [28].

Lefèvre et al. reported median cfDNA decreased from 0.92 ng/µL at baseline to 0.78 ng/µL at MT (*p* < 0.01). Changes in cfDNA levels during CRT were not associated with prognosis [27].

In the CTC study by Ruano et al., mid-treatment levels were not reported [13].

### 3.5. End-of-Treatment and Surveillance CBs and Outcomes (Table 3a–c)

Across studies using viral HPV or tumor-informed ctDNA assays, EOT and surveillance ctDNA measurements were strongly associated with recurrence risk and durability of response following treatment; studies evaluating non-specific cfDNA did not report similar outcome correlations.

In Bercz et al., 6/73 (8%) had persistent ctDNA post CRT; these patients had 61% locoregional failure and one-year PFS of 44%. The ctDNA assay had 100% sensitivity for recurrence in surveillance, predating clinical or radiographic recurrence in all cases [21].

Alvarez et al. reported that ctDNA decreased from 23 to 0.01 MTM/mL (*p* = 0.01), and 95% of patients cleared ctDNA by treatment completion, with molecular clearance occurring approximately 100 days before clinical response [20].

Azzi et al. did not report EOT numerical ctDNA data; however, most stage I–III patients who remained disease-free achieved ctDNA clearance following CRT. In the curative-intent cohort (stage I–III, n = 30), 27 patients were evaluable after excluding three with early recurrence or missing post-therapy testing. Among these, 23 were ctDNA-negative and 4 were ctDNA-positive after definitive therapy. Of 23 ctDNA-negative patients, only one had biopsy-proven residual disease (NPV 95.7%), while three of four ctDNA-positive patients developed disease progression, and one had a partial radiologic response. Post-treatment ctDNA positivity was strongly associated with inferior DFS (median 11.4 months vs. not reached; HR 28.0, 95% CI 2.8–285.0; *p* = 0.005). When DFS was redefined as first radiologic or molecular recurrence, ctDNA positivity remained highly prognostic (HR 109.6; *p* < 0.0001). In one case, ctDNA elevation preceded radiologic recurrence by approximately 200 days [12].

Agarwal et al. noted 4/11 patients with residual/recurrent disease in the series. Three of the four cases that had residual disease/recurrence failed to clear ctDNA by EOT with the NavDx (TTMV-HPV DNA) assay, suggesting an observed sensitivity of 75% for the test to detect recurrence at this timepoint within this small series [18].

In Kabarriti’s study, 19/25 (76%) baseline-positive patients cleared TTMV-HPV DNA during or within 3 months of CRT, translating to significantly better recurrence-free survival than those who did not (*p* = 0.0099). They also observed that during post-treatment surveillance, 104 of 117 patients (88.9%) underwent TTMV-HPV DNA testing, of whom 22 (21.2%) had at least one positive result. Clinical recurrence occurred in 27 patients (26%), including 14 local and 13 distant failures. Positive post-treatment samples showed a median TTMV-HPV DNA score of 112 (range 4–44,185). On a per-patient basis, the authors report that post-treatment testing demonstrated a sensitivity of 82.8%, specificity of 98.4%, positive predictive value (PPV) of 96.0%, and negative predictive value (NPV) of 92.5%; per-test sensitivity and specificity were 85.4% and 99.3%, respectively (PPV 97.6%, NPV 95.0%). When analysis was restricted to patients who were baseline-positive for TTMV-HPV DNA, sensitivity rose to 90% with 100% specificity and PPV and NPV of 94.7%. ctDNA positivity preceded clinical or radiologic recurrence in 58.3% of cases, with a median lead time of 59 days (range 10–536). TTMV-HPV DNA testing resolved 94.3% of cases with indeterminate imaging or clinical findings [17].

Cabel et al. noted that at EOT, ctDNA was detectable in 3 of 18 patients (17%), all of whom developed early metastatic relapse at 2.9, 3.8, and 4.3 months post treatment. Among the 15 patients who were ctDNA-negative at EOT, only one (7%) experienced a local recurrence at 8.5 months. The presence of residual ctDNA at EOT was strongly associated with shorter DFS (*p* < 0.0001). During surveillance, nine additional samples collected 90–150 days after CRT were all ctDNA-negative, and none of these patients experienced recurrence [23].

In the Lefèvre study, patients with persistent molecular disease (n = 13) had measurable pHPV at EOT and showed no local failures but a 31% rate of distant relapse. Additionally, 22 of 73 patients (30%) had undetectable pHPV initially, among whom 18% (4/22) later developed local recurrence. During surveillance, 41 patients had follow-up samples, and several with persistent pHPV at EOT demonstrated rising levels preceding distant relapse. For patients with distant failures, median EOT pHPV was 0.12%, increasing to 13.96% at recurrence. In isolated cases, molecular recurrence was detected before radiologic evidence, though lead time was not quantified [16].

Lee et al.’s panHPV-detect assay showed that at MT/EOT (6–12 weeks post-CRT), cHPV-DNA became undetectable in 7 of 8 evaluable patients at 6 weeks and in 15 of 17 patients (88%) at 12 weeks. The two patients (12%) who remained ctDNA-positive at 12 weeks both relapsed (one locally and one distantly) resulting in 100% sensitivity and specificity for predicting relapse at this timepoint. All 12 patients who achieved a complete clinical and MRI response were ctDNA-negative, while four of five patients with partial response cleared ctDNA and did not relapse. During surveillance beyond 12 weeks, one patient showed persistent ctDNA elevation despite a complete clinical response and later developed distant relapse at nine months. Conversely, four patients with MRI abnormalities but undetectable ctDNA at 12 weeks remained disease-free on follow-up PET/MRI at 6–12 months [24].

Morris et al. failed to show that EOT ctDNA alone was prognostic (HR 1.6, 95% CI 0.35–7.4, *p* = 0.48). There were 13 patients with positive EOT ctDNA versus 43 patients with negative EOT ctDNA and only 10 total recurrences in the cohort at median follow-up of 16.3 months (IQR 7.1–26.8). During surveillance beyond three months post CRT, ctDNA detection strongly correlated with relapse, occurring in 80% of ctDNA-positive patients compared with 2% of ctDNA-negative patients (OR 168; *p* < 0.0001). Median RFS was 4.9 months for ctDNA-positive versus not reached for ctDNA-negative patients (HR 39.2, 95% CI 4.6–330; *p* < 0.0001); at six months, median RFS was 5.6 months versus not reached (HR 32.0, 95% CI 1.8–560; *p* < 0.0001). Surveillance testing demonstrated 89% sensitivity, 95% specificity, 80% positive predictive value, and 98% negative predictive value. Two patients experienced recurrence despite being ctDNA-negative at three months, both converting to positive at later timepoints [25].

Małusecka et al. reported that cfDNA concentrations peaked at the EOT and subsequently declined during follow-up. Median cfDNA levels decreased from 23.40 ng/mL at end of treatment to 11.44 ng/mL at 24 months and 7.46 ng/mL beyond two years. Across the cohort, cfDNA levels were not associated with treatment response or survival outcomes during follow-up (median follow-up of 30 months) [28].

Lefèvre et al. reported that median cfDNA increased from MT to 0.89 ng/µL at EOT (*p* < 0.01) and subsequently declined to 0.71 ng/µL at one-year follow-up (*p* < 0.01 vs. baseline). Higher cfDNA levels at EOT were associated with more severe radiation dermatitis (1.09 vs. 0.85 ng/µL, *p* = 0.04). cfDNA kinetics during treatment were not associated with recurrence or survival (median follow-up 22 months), although patients who relapsed had baseline cfDNA levels above the 25th percentile (*p* = 0.05) [27].

Lastly, Ruano et al. reported that at the 6–8 week post-treatment assessment, 11/15 patients (73%) achieved complete clinical response. Only one patient developed disease recurrence (median follow-up of 22.2 months), which was associated with a substantial increase in CTC levels. No consistent associations were identified between CTC expression markers and outcomes [13].

## 4. Discussion

The current systematic review highlights ctDNA as a promising biomarker for monitoring treatment response, detecting minimal residual disease, and refining follow-up strategies in ASCC.

Baseline CB detection rates differ depending on cohort selection. In general ASCC populations, HPV-targeted ctDNA is detectable in approximately 59–88% of patients [16,23]. Studies with confirmed HPV-positive tumors (e.g., Lee et al. [24]) or those using cfDNA quantification (e.g., Jakobsen et al. [29]) report 100% baseline positivity. For clarity, “positivity” refers to assay-defined detection and is not directly comparable across platforms. Importantly, universal detectability in cfDNA-based studies reflects the non-specific nature of total circulating DNA and should not be interpreted as viral or tumor-specific ctDNA detection or as evidence of equivalent prognostic value. Generally, more advanced-stage ASCC or nodal involvement correlated with higher rates of detection and higher concentrations of ctDNA. Higher baseline ctDNA levels tended to suggest worse DFS and OS [16]. Overall, results suggest that HPV-targeted ctDNA and tumor-informed NGS assays accurately represent tumor burden at baseline, whereas non-specific cfDNA assays have not shown a reliable correlation and should not currently be considered interchangeable with viral or tumor-informed ctDNA-based approaches in ASCC. Inherent limitations for the different assays exist: HPV-targeted assays are not useful in approximately 10% of ASCC cases that are not HPV-driven and reported sensitivity rates may differ depending on whether an analysis can be restricted to HPV-positive ASCCs confirmed by direct testing of tumor tissue. Tumor-informed NGS assays on the contrary should be applicable to both HPV-driven and non-HPV driven ASCC; however, they require collection and processing of a biopsy which can lead to practical and logistical limitations in some cases. In general, whole-exome tumor-informed NGS demonstrates sensitivity comparable (79% in Bercz et al. [21]) to TTMV-HPV DNA assays (85.4% in Kabarriti et al. [17]) at baseline.

Early ctDNA dynamics as assessed by mid-treatment clearance emerged as an important prognostic biomarker. In Bercz et al.’s mPCR-NGS cohort (n = 88), 53% of baseline-positive patients cleared ctDNA by MT with a corresponding 100% rate of PFS and complete clinical response, whereas persistent ctDNA at this timepoint was associated with higher rates of treatment failure (LRF rate of 26% and 81% 1-year PFS) [21]. Lefèvre et al. [16] similarly showed no treatment failures amongst patients with early clearance. In the study by Agarwal et al. (n = 13), failure to clear ctDNA by week 4 resulted in a 50% rate of treatment failure (3/6 patients) [18]. These findings mirror ctDNA dynamics in response to CRT in the setting of other HPV-driven cancers. Chera et al. previously found that week 4 clearance of TTMV-HPV-DNA was associated with a lower risk of recurrence in patients treated for HPV-associated oropharyngeal cancer [30]. Chemo-immunotherapy induction for localized ASCC has also been shown to lead to high rates of ctDNA clearance (90%) in the absence of radiation [26]. Whether clearance in this setting is transient or sustained remains unknown.

EOT and early surveillance ctDNA measurements consistently differentiated between patients who later recurred and those who remained disease-free across multiple cohorts using viral HPV and tumor-informed ctDNA assays. Persistent ctDNA at EOT was uncommon but strongly associated with subsequent relapse. In Bercz et al., 8% of patients remained ctDNA-positive after CRT; this subgroup had a 61% locoregional failure rate and a one-year PFS of 44%. In all cases of recurrent disease, ctDNA positivity preceded clinical/imaging findings [21]. In the Kabarriti cohort, NavDx testing showed a sensitivity of 85.4%, specificity of 99.3%, PPV of 97.6%, and NPV of 95.0%, with ctDNA often preceding radiologic recurrence by a median of 59 days [17]. Conversely, Morris et al. did not identify EOT as prognostic in their study; however ctDNA detection more than three months after CRT was associated with markedly higher recurrence risk (RFS HR 39.2; *p* < 0.0001), occurring in 80% of ctDNA-positive patients versus 2% of ctDNA-negative patients (OR 168; *p* < 0.0001). Surveillance sensitivity reached 89%, with a specificity of 95% and an NPV of 98% [25].

In Kabarriti et al.’s retrospective cohort, TTMV-HPV DNA testing correctly resolved 94.3% (33/35) of clinically indeterminate findings by predicting recurrence status [17]. The feasibility of ctDNA as a biomarker for treatment modification has been successfully demonstrated in the INTERACT-ION trial which used ctDNA clearance as a criterion for a node-sparing radiation approach [26].

## 5. Limitations and Future Directions

Despite consistent strong associations between ctDNA levels and clinical outcomes, several limitations remain. Most ASCC studies were small, single-institution cohorts with heterogeneous methodologies, non-standardized timepoints, and limited follow-up. Surveillance strategies also varied across studies and were not standardized; follow-up schedules and imaging frequency were inconsistently reported, which may have influenced DFS and PFS estimates. Most cohorts were non-blinded, raising the possibility that CB positivity prompted earlier or more intensive clinical or radiographic evaluation, introducing ascertainment bias. Diverse assay platforms (viral, tumor-informed, CTC and total cfDNA assays) complicate comparisons across studies. In addition, some included studies contained small proportions of metastatic patients within otherwise predominantly localized cohorts, which may limit strict applicability to stage I–III disease; however, findings were interpreted in the context of curative-intent populations where applicable. When reported, ctDNA was most analyzed from plasma; however, several studies did not explicitly specify the biological material. Definitions of molecular clearance varied widely. Given low rates of recurrence and small sample sizes, few conclusions about recurrence patterns with ctDNA monitoring can be drawn. For example, it is not clear if differences in ctDNA sensitivity can detect recurrence in different organ sites in ASCC. From other cancers, ctDNA has generally had less sensitivity to detect lung and peritoneal recurrences [31]. This has not been systematically evaluated in ASCC and represents an important gap for future research. No randomized trials have evaluated whether ctDNA-guided management and surveillance affect survival or assessed impacts of testing on quality of life in localized ASCC. As this review was descriptive and did not involve pooled analyses, a formal sensitivity analysis excluding conference abstracts was not performed; however, findings from conference abstracts were interpreted cautiously given their limited methodological detail. In addition, available studies did not permit meaningful subgroup analyses by patient age, comorbidity burden, or performance status, and HPV-negative ASCC was either absent or underrepresented, limiting conclusions regarding CB performance in these clinically important subpopulations. Finally, most studies focused on the clinical performance of the CB rather than its underlying biological mechanisms or integration with complementary biomarkers such as imaging, tissue genomics, or immune correlates, limiting mechanistic insight into treatment resistance and disease evolution in ASCC.

Future research should incorporate ctDNA into prospective trials using standardized assays and predefined timepoints (baseline, MT, EOT, and surveillance). Despite its rarity, randomized clinical trials are possible in ASCC and should be pursued to further validate the utility of this biomarker in clinical practice. Lessons learned from successes and failures of ctDNA-guided management strategies in other cancer types should be applied. In colorectal cancer, the FIND trial showed that ctDNA-guided surveillance could trigger comprehensive imaging that led to earlier detections amenable to curative-intent therapy (50% vs. 22.6% without ctDNA-guidance; RR 2.21; 95% CI 1.06–4.78; *p* = 0.034) [32]. Curative-intent locoregional therapy is less established as a paradigm for oligometastatic disease in ASCC, though retrospective evidence supports its consideration [33]. It is possible that ctDNA-positive patients at EOT or surveillance may benefit from imaging with short intervals or the use of comprehensive imaging techniques to detect early recurrence.

The success of ctDNA-guided management for ASCC will depend on the sensitivity and specificity of the ctDNA assay and the efficacy of the therapeutic interventions used. Newer generations of tumor-informed ctDNA assays may use whole-genome sequencing rather whole-exome sequencing to improve assay sensitivity [34]. The DYNAMIC II study was able to demonstrate non-inferiority in 2-year DFS with ctDNA-guided management in stage II colon cancer despite nearly halving the rates of adjuvant therapy (15% vs. 28%) [35]; however, non-inferiority was not met for ctDNA-guided de-escalation in stage III colon cancer in the subsequent DYNAMIC III study [36]. This suggests the test used lacked sufficient sensitivity at the decision-making timepoint for adjuvant therapy. The recently published ACT4 (PLATO) phase II trial demonstrated non-inferiority of shortened CRT for T1-2N0 ASCC [37]. It is possible that mid-treatment ctDNA clearance may be an alternative marker for de-escalation, though this requires prospective validation. A similar approach is currently being studied in HPV-driven oropharyngeal SCC (NCT05541016).

The IMvigor011 trial in muscle-invasive bladder cancer represents a landmark example of ctDNA-guided adjuvant therapy with escalation to atezolizumab resulting in an overall survival benefit over placebo for ctDNA-positive patients (HR 0.59, *p* = 0.01) [38]. Efforts of MRD treatment escalation in colorectal cancer have conversely failed, potentially due to modest additional cytotoxic activity of the investigational therapies studied [36,39]. Adjuvant immune-checkpoint inhibition is currently being investigated in ASCC following CRT in patients with stage IIB or greater disease (NCT03233711) and has established efficacy in advanced disease [40,41]. As many patients achieve cure with chemoradiation alone, an unselected approach risks overtreatment.

Using ctDNA could potentially enrich selection of patients most likely to benefit from further treatment following CRT. Morris et al. argue that 3-month ctDNA positivity should be the timepoint for selection [25]. We argue that MT and/or EOT ctDNA positivity is associated with elevated risk of treatment failure and may allow for earlier intervention within a window with the greatest opportunity for success. The outcomes of Bercz et al. [21] and other groups based on ctDNA status at these timepoints can serve as benchmarks to improve upon when designing endpoints for future escalation trials.

## 6. Conclusions

Collectively, the findings from localized ASCC (particularly HPV-associated disease) correspond with those noted in other cancers where CBs have been used to identify patients at elevated risk of recurrence and have been incorporated into treatment strategies in clinical trials. Similar to these settings, ASCC studies show that persistent ctDNA after CRT is associated with early relapse, while sustained molecular clearance is associated with excellent outcomes. These findings highlight the potential for CBs, particularly ctDNA, to support risk stratification and improve disease monitoring, including clarifying indeterminate imaging findings and identifying recurrence earlier than conventional approaches. However, given the heterogeneity in assay platforms, study design, and outcome definitions, as well as the absence of randomized data, these observations should be interpreted with caution. The application of ctDNA to guide escalation or de-escalation strategies in ASCC remains investigational. Prospective, randomized studies with longer follow-up are needed to establish standardized thresholds, validate clinically actionable timepoints, and determine whether ctDNA-directed management can improve outcomes or reduce treatment-related burden in patients with ASCC.

## Data Availability

The original contributions presented in this study are included in the article. Further inquiries can be directed to the corresponding author.

## Supplementary Materials

The following supporting information can be downloaded at: https://www.mdpi.com/article/10.3390/cancers18101626/s1, Table S1: Risk of Bias assessment—Newcastle–Ottawa scale; Table S2: Summary matrix.
