# Supplementary material for: Circulating Biomarkers in Localized Anal Squamous Cell Carcinoma Across Treatment Timepoints: A Systematic Review
Source: Cancers (Basel). 2026 May 18;18(10):1626. doi: 10.3390/cancers18101626 (PMC13204479; doi:10.3390/cancers18101626)
Supplement: Supplementary file 1 [file cancers-18-01626-s001.zip › Supplementary Table S1. Risk of Bias assessment - NOS.pdf]

Circulating Biomarkers in Localized Anal Squamous Cell Carcinoma Across Treatment Timepoints: A Systematic Review

Oluwatayo Adeoye[1], Abdulsabur Sanni[2], Khujasta Gul[1], Jakob Hamilton[3], Ahmed Abdelhakeem[1], Michael Rutenberg[4], Zhaohui Jin[5], Umair Majeed[1], Jeremy Jones[1], Conor O'Donnell[1]

[1]Department of Hematology/Oncology, Mayo Clinic Jacksonville

[2]Department of Hematology/Oncology, University of Minnesota

[3]Department of Internal Medicine, Mayo Clinic Jacksonville

[4]Department of Radiation Oncology, Mayo Clinic Jacksonville

[5]Department of Oncology, Mayo Clinic Rochester

Supplementary Table S1. Risk of Bias assessment - Newcastle-Ottawa scale

Study (Author, Year)	S1	S2	S3	S4	C1	C2	O1	O2	O3	Selection subtotal (0-4)	Comparability subtotal (0-2)	Outcome subtotal (0-3)	Total Stars (0-9)	Risk of Bias
Jakobsen et al., 2025 (cfDNA DFA, Denmark, n=126)	*	/	*	*	*	/	*	*	*	3	1	3	7	Low
Azzi et al., 2023 (Signatera, US, n=251/37 analyzed)	*	/	*	*	*	/	*	*	/	3	1	2	6	Moderate
Kabarriti et al., 2025 (NavDx, 7 centers, n=117)	*	/	*	*	*	/	*	*	*	3	1	3	7	Low
Agarwal et al., 2025 (NavDx, US/Mayo FL, n=13)	*	/	*	*	/	*	*	*	*	3	1	3	7	Low
Bercz et al., 2025 (Signatera, MSKCC, prospective, n=88)	*	/	*	*	*	/	*	*	*	3	1	3	7	Low
Alvarez et al., 2023 (Signatera, US, prospective, n=41)	*	/	*	*	/	/	*	/	/	3	0	1	4	Moderate
Cabel et al., 2018 (ddPCR HPV16/18, France, LA-ASCC, n=33)	*	/	*	*	*	/	*	*	*	3	1	3	7	Low
Lee et al., 2020 (panHPV-detect NGS, UK, n=24)	*	/	*	*	*	/	*	*	*	3	1	3	7	Low
Małusecka et al., 2022 (qPCR cfDNA TERT, Poland, n=26)	*	/	*	*	/	/	*	*	*	3	0	3	6	Moderate
Lefèvre et al., 2020 (DFA cfDNA, Denmark, n=80)	*	/	*	*	*	/	*	*	*	3	1	3	7	Low
Lefèvre et al., 2021 (ddPCR pHPV kinetics, Denmark, n=88)	*	/	*	*	*	/	*	*	*	3	1	3	7	Low
Mazurek et al., 2023 (qPCR ctHPV16, Poland, n=62)	*	/	*	*	*	/	*	*	*	3	1	3	7	Low
Ruano et al., 2023 (CTCs + HPV CISH, Brazil, n=15)	*	/	*	*	/	/	*	*	/	3	0	2	5	Moderate
Morris et al., 2025 (ddPCR 13 HR-HPV types, MDACC, n=65)	*	/	*	*	*	/	*	*	*	3	1	3	7	Low
Kim et al., 2025 (dd PCR, France, n = 55)	*	/	*	*	*	/	*	*	*	3	1	3	7	Low

Selection (S1-S4): S1, Representativeness; S2, Non-exposed cohort; S3, Ascertainment of exposure (assay validation); S4, Outcome absent at start
Comparability (C1-C2): C1, Control of key confounders; C2, Additional factors
Outcome (O1-O3): O1, Objective outcome assessment; O2, Follow-up long enough (≥12 mo); O3, Adequate follow-up (≥80% or described)
*Higher scores indicate lower risk of bias.
