# Supplementary material for: Circulating Biomarkers in Localized Anal Squamous Cell Carcinoma Across Treatment Timepoints: A Systematic Review
Source: Cancers (Basel). 2026 May 18;18(10):1626. doi: 10.3390/cancers18101626 (PMC13204479; doi:10.3390/cancers18101626)
Supplement: Supplementary file 1 [file cancers-18-01626-s001.zip › Supplementary Table S2. Summary Matrix.pdf]

Supplementary Table S2. Summary Matrix

Study	N	Sex M/F	HPV/p16 status	Assay type	Positivity/biomarker definition	Baseline	MT	EOT/Surveillance	Study summary
**Cabel et al., 2018**	33	4/29	HPV16/18+ 31/2	HPV16/18 ddPCR	≥3 droplets at control amplitude; copies/mL	Yes	No	Yes	Post-CRT positivity strongly associated with shorter DFS; all 3 EOT-positive patients relapsed early. Baseline positivity was not prognostic.
**Lee et al., 2020**	24 enrolled; 21 baseline; 17 paired	9/12 among baseline cohort	HPV+ 20/21; HPV- 1/21	panHPV-detect targeted NGS	≥5.5 HPV amplicons with >10 reads	Yes	Yes	Yes	Baseline sensitivity/specificity 100% in HPV+ ASCC; 12-week positivity in 2/17, both relapsed.
**Lefèvre et al., 2020**	80	19/61	p16+ 55/62; p16− 7/62	Total cfDNA DFA	Quantitative serum cfDNA ng/µL; no binary tumor-specific cutoff	Yes	Yes	Yes	cfDNA correlated with tumor burden and decreased during therapy; baseline above 25th percentile associated with relapse risk, but marker was non-tumor-specific.
**Lefèvre et al., 2021**	88	23/65	p16+ 72/88; p16− 13/88;	Multiplex HPV ddPCR	pHPV as % of total cfDNA; kinetic categories	Yes	Yes	Yes	Fast clearance by MT had no failures; slow clearance linked

			unknown 3						to local failure; persistent EOT pHPV linked to distant relapse.
**Małusecka et al., 2022**	26	5/21	Not uniformly reported	qPCR total cfDNA using TERT	Quantitative total cfDNA ng/mL; not tumor-specific	Yes	Yes	Yes	cfDNA peaked at EOT and correlated with tumor volume/neutrophils, but did not correlate with response or survival.
**Alvarez et al., 2023**	41	NR	NR	Tumor-informed mPCR-NGS, Signatera	MTM/mL; tumor-bespoke ctDNA	Yes	Yes	Yes	ctDNA cleared earlier than cCR, median 31 vs 131 days. Two patients converted from negative to positive during follow-up before clinical/radiographic relapse; longer follow-up needed.
**Azzi et al., 2023**	251 total; 37 annotated; 27 curative-intent outcome cohort	Full cohort 71/180; annotated 18/19	Annotated HPV+ 20/21; HPV− 1/21	Tumor-informed mPCR-NGS, Signatera	≥2 tumor-specific SNVs detected; MTM/mL	No	Yes	Yes	Post-definitive therapy ctDNA positivity associated with shorter DFS in stage I-III disease; HR 28.0, p=.005.

**Mazurek et al., 2023**	62	13/49	HPV16 assay; tumor HPV not all specified	HPV16 qPCR	HPV16 copies/mL plasma; log10 viral load	Yes	Yes	Yes	Decline to undetectable ctHPV16 correlated with remission; persistence/re-emergence associated with progression or relapse.
**Ruano et al., 2023**	15	5/10	Tumor p16+ 15/15; HPV DNA in CTCs 14/15	CTC isolation + HPV CISH	CTCs/mL and HPV DNA in CTCs	Yes	No	No	Feasibility study; HPV-positive CTCs common at baseline and post-treatment, but outcome correlations limited by small sample size.
**Kabarriti et al., 2025**	117	32/85	HPV-driven ASCC; HPV16 74/81 typed	NavDx TTMV-HPV DNA ddPCR	Positive/negative/indeterminate; TTMV-HPV DNA score	Yes	No	Yes	Post-treatment sensitivity 82.8%, specificity 98.4%, PPV 96.0%, NPV 92.5%; median lead time 59 days.
**Agarwal et al., 2025**	13; 11 analyzed	NR	HPV+ and baseline ctDNA+ 11/13	NavDx TTMV-HPV DNA ddPCR	Detectable vs undetectable; copies/mL not fully reported	Yes	Yes	Yes	Week-4 and EOT clearance associated with favorable outcomes; 3/4 residual/recurrent cases had not cleared by EOT.

**Bercz et al., 2025**	88	NR	NR	Tumor-informed mPCR-NGS, Signatera	Positive/negative tumor-informed ctDNA	Yes	Yes	Yes	MT negativity associated with 0% LRF and 1-year PFS 100%; post-treatment positivity associated with 61% LRF and 1-year PFS 44%.
**Jakobsen et al., 2025**	126	34/92	p16+ 113/126; p16– 13/126	Total cfDNA DFA	Quantitative serum cfDNA ng/μL; percentage decline; no tumor-specific cutoff	Yes	Yes	No	Lower cfDNA decline during CRT associated with nonresponse/failure; failure to fall below baseline 75th percentile associated with worse DFS.
**Morris et al., 2025**	65	15/50	HPV+ 51/52 tested	Multiplex HPV ddPCR	≥16 HPV copies/mL positive	Yes	No	Yes	EOT positivity was not prognostic, but HPV ctDNA ≥3 months post-CRT strongly predicted recurrence; HR for RFS 39.2.
**Kim et al., 2025, INTERACT-ION**	55 enrolled; 54 evaluable	13/42	HPV16 90%; baseline HPV ctDNA+ 40/54	HPV digital PCR	BCR: >20 copies/mL to undetectable	Yes	Yes	Yes	HPV ctDNA clearance was incorporated as part of response-adapted treatment selection; 36/40 baseline-positive

				patients achieved BCR

Footnote: This table represents a narrative summary of included studies. Given heterogeneity in assay platforms, biomarker definitions, sampling timepoints, and clinical endpoints (e.g., DFS, RFS, PFS, cCR), results were not pooled and no cross-study quantitative comparisons were performed. "Yes" indicates that the given timepoint was reported in the study; "No" indicates that it was not reported.

Abbreviations: ASCC, anal squamous cell carcinoma; BCR, biological complete response; cfDNA, cell-free DNA; ctDNA, circulating tumor DNA; CTC, circulating tumor cell; ddPCR, droplet digital polymerase chain reaction; DFA, DNA fluorescence assay; HPV, human papillomavirus; HR-HPV, high-risk human papillomavirus; IQR, interquartile range; NGS, next-generation sequencing; NR, not reported; p16, cyclin-dependent kinase inhibitor 2A; qPCR, quantitative polymerase chain reaction; TERT, telomerase reverse transcriptase; TTMV-HPV DNA, tumor tissue-modified viral human papillomavirus DNA; MT, mid-treatment; EOT, end of treatment; RECIST, Response Evaluation Criteria in Solid Tumors; INRT, involved-node radiotherapy; DFS, disease-free survival; PFS, progression-free survival; PPV, positive predictive value; NPV, negative predictive value.
